# Comparison of Human Milk Immunoglobulin Survival during Gastric Digestion between Preterm and Term Infants

**DOI:** 10.3390/nu10050631

**Published:** 2018-05-17

**Authors:** Veronique Demers-Mathieu, Mark A. Underwood, Robert L. Beverly, Søren D. Nielsen, David C. Dallas

**Affiliations:** 1 Nutrition Program, School of Biological and Population Health Sciences, College of Public Health and Human Sciences, Oregon State University, Corvallis, OR 97331, USA; Veronique.Demers-Mathieu@oregonstate.edu (V.D.-M.); beverlyr@oregonstate.edu (R.L.B.); sodn@food.au.dk (S.D.N.); 2 Department of Pediatrics, University of California, Davis, Sacramento, CA 95817, USA; munderwood@ucdavis.edu

**Keywords:** passive immunity, antibodies, lactation, peptidomics, prematurity, proteolysis, breast milk

## Abstract

Human milk provides immunoglobulins (Igs) that supplement the passive immune system of neonates; however, the extent of survival of these Igs during gastric digestion and whether this differs between preterm and term infants remains unknown. Human milk, and infant gastric samples at 2 h post-ingestion were collected from 15 preterm (23–32 week gestational age (GA)) mother-infant pairs and from 8 term (38–40 week of GA) mother-infant pairs within 7–98 days postnatal age. Samples were analyzed via ELISA for concentration of total IgA (secretory IgA (SIgA)/IgA), total secretory component (SC/SIgA/SIgM), total IgM (SIgM/IgM), and IgG as well as peptidomics. Total IgA concentration decreased by 60% from human milk to the preterm infant stomach and decreased by 48% in the term infant stomach. Total IgM and IgG concentrations decreased by 33% and 77%, respectively, from human milk to the term infant stomach but were stable in the preterm infant stomach. Release of peptides from all Ig isotypes in the term infant stomach was higher than in the preterm stomach. Overall, the stability of human milk Igs during gastric digestion is higher in preterm infant than in term infants, which could be beneficial for assisting the preterm infants’ immature immune system.

## 1. Introduction

Immunoglobulins (Igs) are important effectors of the adaptive immune system [1]. During the third trimester, the mother’s placenta transports IgG to the fetus via a neonatal Fc receptor. These maternal IgG antibodies protect the infant during the first 6 months of postnatal age while the infant’s own immune system is developing [2]. After birth, human milk provides another form of protection against pathogens for infants, as it contains an array of Igs, including IgA, secretory IgA (SIgA), IgM, secretory IgM (SIgM), and IgG [3,4]. Indeed, feeding mother’s milk reduces risks of infectious diseases in the respiratory and gastrointestinal tract in infancy [5]. Though human milk provides different Ig isotypes, milk SIgA is thought to be the most important in the infant gut as it neutralizes bacterial and viral pathogens by binding to them, thus reducing their ability to interact with epithelial cells and infect [6,7]. The presence of SIgA in human milk temporarily replaces the normal intestinal SIgA secretion that is lacking in the infant until 4 weeks of postnatal age [8].

SIgA is the first line of defense in protecting the intestinal epithelium from pathogens by immune exclusion [9]. Though SIgA is the most well-known and abundant Ig of intestinal secretions [10], IgG and IgM are also secreted by plasma cells in neonatal intestinal mucosa [11,12]. IgG may have function in the gut: passive administration of virus-neutralizing IgG prevented mucosal immunodeficiency virus transmission from mother to infant in macaques [13], suggesting potential transmission of these IgG in the gut. However, the ability of IgG to bind to viruses to prevent attachment to the mucosal surface or trap pathogens in mucus appears to be inefficient compared with SIgA [13]. Human neonatal Fc receptor (FcRn) may be able to transport IgG (alone or bound to an antigen) across the intestinal epithelial barrier into the lamina propria [11]. Though IgM-secreting cells have been identified in the infant gut [12] and IgM was shown to be synthesized in amounts similar to IgA in pig small intestinal mucosal explants [14,15], no study has determined the extent to which IgM plays a role in the adult or infant intestinal mucosal immune defense.

In order to function in immunoprotection in the gut, IgA and potentially the other Ig must resist proteolytic degradation and remain intact and able to bind to pathogens through the digestive system. Though the provision of milk Igs to the infant is known to reduce infection risk, the degree to which Igs survive in the gastrointestinal tract remains unknown. Few studies have investigated the stability of Igs in digestion. Two oral supplementation studies (in adults fed bovine colostrum SIgA/IgA, IgM and IgG [16] and in preterm infants fed serum IgA and IgG [17]) demonstrated that IgG and IgM survive intact to the stool, whereas SIgA/IgA does not. However, some studies have demonstrated that human milk-derived SIgA survived intact to the infant stool and urine [8,18,19].

Our previous study demonstrated that preterm infants had lower gastric digestion capacity for human milk proteins than term infants [20]. This difference in digestion capacity could affect the survival of immunoglobulins. Our recent studies demonstrated that preterm infants partially degrade IgA but not IgG and IgM in the stomach [21,22]. Whether differences in preterm and term infant gastric digestion result in differences in Ig survival remain unknown. The aim of the present study was to determine whether preterm infant Ig survival in the stomach is higher than in term infants.

As preterm infants are born early and miss some of the placenta-fetal IgG transfer [23], produce less diverse of antibodies in their gut in comparison with term infants [24], and are at higher risk for bacterial [25] and viral infections [26] than term infants, the presence of Ig in human milk may be even more critically important to their health outcomes than for term infants. Therefore, milk-provided Ig may be even more critically important than for term infants. Increased survival of milk Igs in the preterm infant stomach due to lower protein digestion capacity could provide increased direct support of their naïve immune systems through immune exclusion.

## 2. Materials and Methods

### 2.1. Participants and Sample Collection

This study was approved by the Institutional Review Board of the University of California, Davis (UC Davis) and Oregon State University (OSU). Inclusion criteria included inpatient admission to the neonatal intensive care unit (NICU), an indwelling nasogastric or orogastric feeding tube and tolerance of full enteral feeding. Most of the enrolled infants required a feeding tube because of uncoordinated or immature capacity to suck and swallow. Exclusion criteria were anatomic or functional gastrointestinal disorders. Enrolled infants had a variety of medical conditions, including respiratory distress syndrome, chronic lung disease, apnea of prematurity, and Dandy–Walker malformation in the premature infants and cleft palate, respiratory distress syndrome, hypoxic-ischemic encephalopathy, and congenital diaphragmatic hernia in the term infants, but no overt gastrointestinal tract issues. Respiratory distress syndrome and congenital diaphragmatic hernia can delay gastric emptying in preterm infants [27,28]; however, the other medical conditions in this cohort have not been associated with an effect on gastric emptying or digestion capacity. Hypoxic-ischemic encephalopathy may result in gastrointestinal tissue damage [29]; however, the effects on gastric emptying and protein digestion are unknown. The infants with hypoxic ischemic encephalopathy were fasted and underwent whole body cooling for 72 h starting shortly after birth, however this treatment was completed and the infants advanced to full enteral feeding prior to enrollment. The infant with congenital diaphragmatic hernia had the defect repaired and was advanced to full enteral feeding prior to enrollment. Gastroesophageal reflux is almost universal in this population; however, none of the infants sampled received medications known to affect gastric pH or gastric digestion capacity, including prokinetics, H2 blockers/antagonists or proton-pump inhibitors. The enrolled infants were clinically stable at the time of sample collection (not on mechanical ventilation, stable vital signs). Samples were collected from 15 premature-delivering mother-infant pairs ranging in gestational age (GA) at birth from 23 to 32 weeks and 8 term-delivering mother-infant pairs ranging in GA at birth from 38 to 40 weeks (Table 1) over 7–98 days of postnatal age at the UC Davis Children’s Hospital NICU in Sacramento, California. Enrolled infants had a variety of medical conditions, but no overt gastrointestinal tract issues or other medical conditions that have been associated with an effect on gastric emptying or digestion capacity. The enrolled infants were clinically stable at the time of sample collection (not on mechanical ventilation, stable vital signs). Human milk samples were collected as described in our previous study [30]. The preterm infants were fed their mother’s milk (raw, not pasteurized) with fortifier (Similac Human Milk Fortifier Powder, Abbott Park, IL). The powdered fortifier contained intact bovine milk proteins and its protein composition was designed to match the whey:casein of human milk (60:40) using non-fat milk and whey protein concentrate. Each 25 mL of human milk was fortified with 0.25 g of bovine milk proteins (adding 10 mg protein/mL). Term infants were fed their mother’s milk (raw, not pasteurized) without fortification. The human milk feedings were delivered via the nasogastric tubes over 30 min. Two hours after the initiation of feeding, 2 mL of each preterm and term infant’s gastric contents were collected in a syringe back through the feeding tube via suction as previously described in our studies [21]. Gastric samples were aspirated at 2 h postprandial to obtain samples that represented a compromise between when adequate sample remained recoverable from the stomach and a maximum feasible length of gastric digestion time had passed based on gastric emptying times [31]. Human milk and gastric samples were placed into sterile vials and stored at −20 °C and were transported to OSU on dry ice and stored at −80 °C.

### 2.2. Sample Reparation and ELISAs

Samples were thawed at 4 °C, pH was determined, and samples were centrifuged at 4226× *g* for 10 min at 4 °C. The infranate was collected, separated into aliquots and stored at −80 °C. The pH of the samples was measured with an S220 SevenCompact pH/Ion meter (Mettler-Toledo, Columbus, OH, USA) equipped with a combined sealed glass electrode.

The spectrophotometric ELISAs were recorded with a microplate reader (Spectramax M2, Molecular Devices, Sunnyvale, CA, USA) with two replicates of blanks, standards, and samples. SoftMax Pro 7.0 Microplate Data Analysis Software (Molecular Devices) was used to create a standard curve with a Four Parameters Logistic curve fit. ELISAs were performed according to the methods described by the manufacturers with some modifications as described (Table S1). The specific Ig concentrations in the samples were determined with antibody specificities as follows: human anti-alpha-chain antibody for total IgA (SIgA/IgA), anti-SC antibody for total SC (SC/SIgA/SIgM), gamma-chain antibody for IgG and mu-chain antibody for total IgM (SIgM/IgM). Concentrations of total IgA, total SC, total IgM and IgG were determined in human milk and gastric samples as well as in the fortifier alone.

For a separate test of the effect of the gastric pH change on Igs, ELISA were performed on standard Igs (total IgA, total SC, total IgM and IgG) in milk before and after incubation at pH 4.5 for 1 h at 37 °C. HCl (10 mM) was used to adjust the pH to 4.5.

### 2.3. Peptidomic Analysis

Peptide extraction from human milk and gastric samples was performed as described previously [32]. Mass spectrometric parameters were as described previously [33]. Spectra were analyzed by database searching in Thermo Proteome Discoverer (v2.1.0.81) using an in-house human milk protein sequence database. The tandem spectra were used to determine the counts and abundance of Ig alpha-chain (from IgA or SIgA), Ig gamma-chain (from IgG), Ig mu-chain (from IgM or SIgM), Ig J-chain (from IgA, SIgA, IgM or SIgM), Ig kappa-chain and Ig lambda-chain (from IgA, SIgA, IgM, SIgM or IgG), SC (f19–603 of total polymeric immunoglobulin receptor (PIgR)), and neonatal Fc receptors (FcRn). Only peptides identified with high confidence (*p* < 0.01) were included, and peptide sequences with multiple modifications were grouped into a single peptide for counts. Peptide counts measured the number of unique peptides identified in a sample whereas peptide abundance measured the ion intensity of the peak in a sample.

### 2.4. Statistical Analyses

Wilcoxon matched-pairs signed-rank test for paired sample comparisons (across milk and gastric samples within the same infant) and Mann–Whitney tests for unpaired sample comparisons (preterm versus term infants) were applied using GraphPad Prism software (version 7.03). All tests were nonparametric as some of the values did not pass the D ’Agostino & Pearson normality test. Linear regression models were applied to determine if the concentrations of total IgA, total SC, total IgM and IgG in human milk and in gastric samples in both infant groups changed across postnatal age, GA, postmenstrual age (PMA), body weight at birth (BW_b_), body weight at sampling (BW_s_), and feed volume. Differences were designated significant at *p* < 0.05. Pearson correlation coefficients (r) were determined when *p* < 0.1. The sample size of preterm (*n* = 15) and term (*n* = 8) paired milk and gastric samples was selected based on our previous study [20] and proved to be adequately powered to detect differences based on the results.

## 3. Results

### 3.1. Infant Demographics

Demographic details for the preterm- and term-delivering mother–infant pairs are presented in Table 1.

### 3.2. Ig Concentrations

#### 3.2.1. Total IgA Concentration

**Figure 1:**
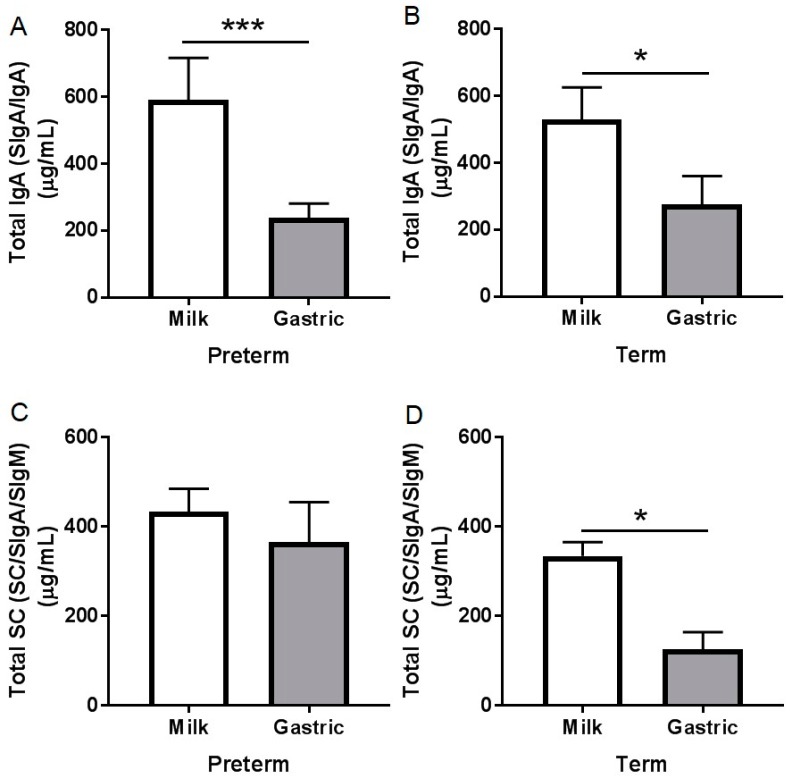
Immunoglobulin concentrations in human milk and gastric samples at 2 h postprandial time from paired mother-infant delivered prematurely (23–32 week of gestational age (GA), 7–98 days of postnatal age) and at term (38–40 week of GA, 16–42 days of postnatal age). Concentration of total IgA (SIgA/IgA) (**A**) in preterm infant samples and (**B**) in term infant samples; Concentration of total secretory component (SC/SIgA/SIgM) (**C**) in preterm infant samples and (**D**) in term infant samples. Values are mean ± SEM, *n* = 15 for preterm infants and *n* = 8 for term infants. Asterisks show statistical significant differences between variables (*** *p* < 0.001; * *p* < 0.05) using the Wilcoxon matched-pairs signed-rank test.

Total IgA (SIgA/IgA) concentration decreased 60% (*p* = 0.001, Figure 1A) from human milk to the preterm infant stomach, and 47.8% from human milk to the term infant stomach (*p* = 0.016, Figure 1B). Total IgA concentration was similar between preterm and term milks and between preterm and term gastric contents (*p* > 0.05, Table S2). Total IgA concentration in preterm milk and gastric samples decreased with increased postnatal age, PMA, and BW_s_ (*p* < 0.05) but did not change in term samples (*p* > 0.05) (Table S3). Total IgA concentration in human milk or gastric contents did not change across GA and feed volume within preterm and term infants (*p* > 0.05, Table S3). When human milk was incubated under in vitro acidic conditions (pH 4.5) to match stomach conditions but without the proteases, there was no decrease in IgA concentration. No IgA was detected in the fortifier.

#### 3.2.2. Total SC Concentration

Human milk total SC (SC/SIgA/SIgM) concentration significantly decreased 62% (*p* = 0.031, Figure 1D) from human milk to the term stomach but did not change in the preterm infant stomach (*p* = 0.33, Figure 1C). Total SC concentration did not differ between human milk from preterm- and term-delivering mothers (*p* = 0.22), nor between preterm and term gastric contents (*p* = 0.11). Total SC concentration in milk and gastric samples in term and preterm infants did not change across GA, postnatal age, or PMA (*p* > 0.05, Table S3). No total SC was detected in the fortifier.

#### 3.2.3. Total IgM Concentration

**Figure 2:**
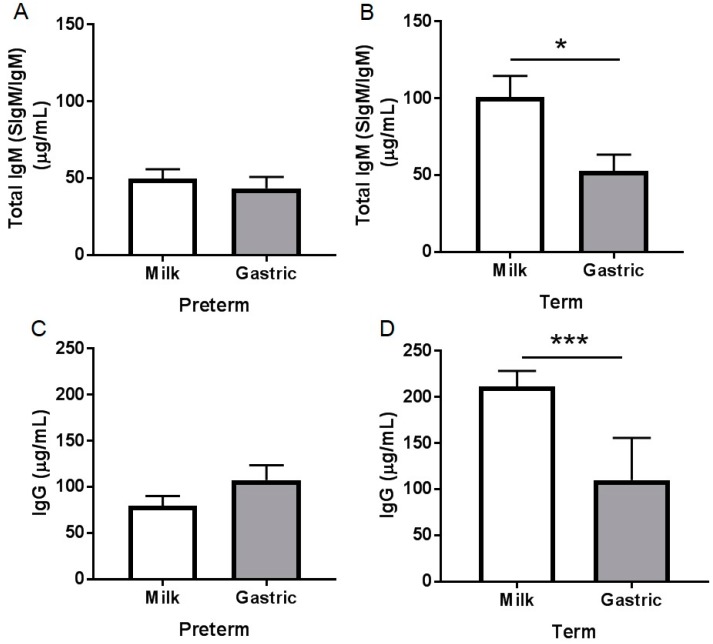
Immunoglobulin concentrations in human milk and gastric samples at 2 h postprandial time from paired mother-infant delivered prematurely (23–32 week of gestational age (GA), 7–98 days of postnatal age) and at term (38–40 week of GA, 16–42 days of postnatal age). Concentration of total IgM (SIgM/IgM) (**A**) in preterm infant samples and (**B**) in term infant samples; Concentration of IgG (**C**) in preterm infant samples and (**D**) in term infant samples. Values are mean ± SEM, *n* = 15 for preterm infants and *n* = 8 for term infants. Asterisks show statistical significant differences between variables (*** *p* < 0.001; * *p* < 0.05) using the Wilcoxon matched-pairs signed-rank test.

Total IgM (SIgM/IgM) concentration decreased significantly (*p* = 0.016, Figure 2B) 33% from human milk to the term infant stomach but did not change in the preterm infant stomach (*p* = 0.54, Figure 2A). Total IgM concentration in human milk from preterm-delivering mothers was 77% lower than in milks from term-delivering mothers (*p* < 0.001, Figure S1A) but did not differ in the gastric contents between preterm and term infants (*p* = 0.56). Total IgM concentration in either milk or gastric samples did not change across postnatal age, GA, PMA, or BW_b_ for term or preterm infants (*p* > 0.05, Table S3). Total IgM concentration decreased in preterm milk with increasing postnatal age or BW_s_ (*p* < 0.05) but did not change in term milk or in either preterm or term stomach samples (*p* > 0.05, Table S3). Total IgM concentration increased with increasing feed volume in preterm milk and gastric samples but did not change in term samples. No total IgM was detected in the fortifier.

#### 3.2.4. IgG Concentration

IgG concentration decreased significantly (48%, *p* = 0.026, Figure 2D) from human milk to the term infant stomach but did not change in the preterm infant stomach (*p* = 0.11, Figure 2C). IgG concentration in human milk from preterm-delivering mothers was 97% lower than in milks from term-delivering mothers (*p* < 0.001, Figure S1B) but did not differ in the gastric contents between preterm and term infants (*p* = 0.58). IgG concentration in preterm and term milk did not change across postnatal age, GA, PMA, BW_b_, or feed volume (*p* > 0.05, Table S3). IgG concentration in preterm gastric samples decreased with increasing PMA or BW_s_ and increased with increasing BW_b_ in term stomach but did not change across GA or feed volume. No IgG was detected in the fortifier.

### 3.3. Peptidomic Results

No peptides (counts or abundance) for Ig alpha-chain, Ig gamma-chain, Ig mu-chain, Ig J-chain, Ig lambda-chain, and Ig kappa-chain were detected in human milk from either the mothers who delivered prematurely or at term, but peptides from each of these proteins appeared in the gastric samples from both preterm and term infants (Figures S2–S4). Peptides from SC (f19–603 of PIgR) was detected in both milk and gastric samples from both preterm and term infants.

**Figure 3:**
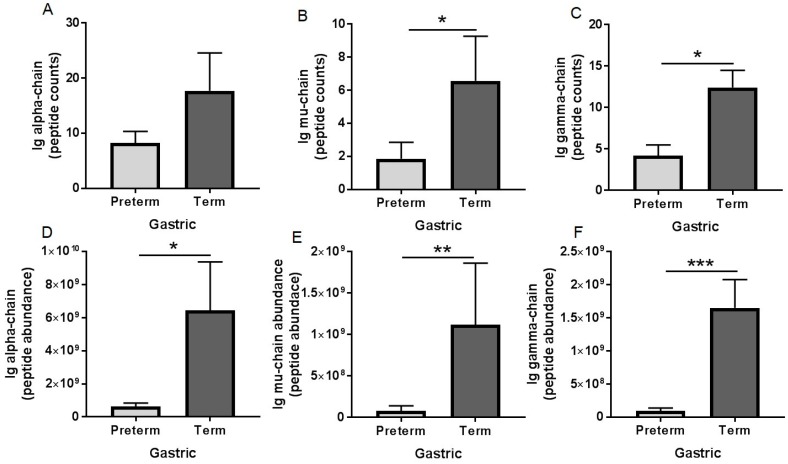
Peptide counts and abundance of human immunoglobulin fragments in human milk and gastric samples at 2 h postprandial time from paired mother-infant delivered prematurely (23–32 week of gestational age (GA), 7–98 days of postnatal age) and at term (38–40 week of GA, 16–42 days of postnatal age). (**A**,**D**) Ig alpha-chain (from SIgA/IgA) and (**B**,**E**) Ig mu-chain (from SIgM/IgM); (**C**,**F**) Ig gamma-chain (from IgG). Values are mean ± SEM, *n* = 15 for preterm infants and *n* = 8 for term infants. Asterisks show statistical significant differences between variables (*** *p* < 0.001; ** *p* < 0.01; * *p* < 0.05) using the Mann–Whitney test (unpaired samples).

**Figure 4:**
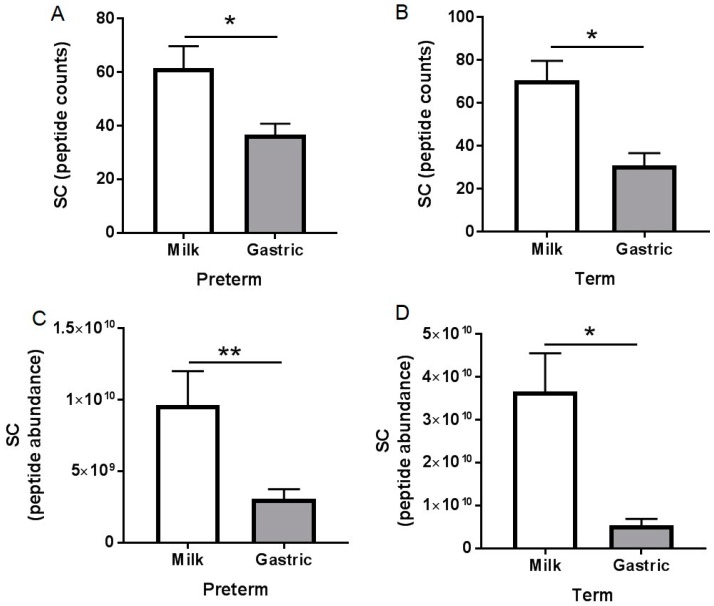
Peptides counts and abundance of SC (f19–603 of PIgR) in human milk and gastric samples from mother–infant pairs with preterm delivery (23–32 week of gestational age (GA), 7–98 days of postnatal age) and term delivery (38–40 week of GA, 16–42 days of postnatal age. Peptides counts of SC (**A**) in preterm infant samples; (**B**) in term infant samples. Peptide abundance of SC (**C**) in preterm infant samples and (**D**) in term infant samples. Values are mean ± SEM, *n* = 15 for preterm infants and *n* = 8 for term infants. Asterisks show statistical significant differences between variables (** *p* < 0.01; * *p* < 0.05) using the Wilcoxon matched-pairs signed-rank test.

Ig alpha-chain peptide counts in gastric samples were 1.2-fold lower in preterm infants than in term infants (not significant, but a tendency: *p* = 0.081, Figure 3A). Ig alpha-chain peptide abundance in gastric contents was 16-fold lower in preterm infants than in term infants (*p* = 0.038, Figure 3D). Ig mu-chain peptide counts and abundance in gastric contents were 2.3- (*p* = 0.032, Figure 3B) and 13-fold (*p* = 0.010, Figure 3E), respectively, lower in preterm infants than in term infants. Ig gamma-chain peptide counts and abundance in gastric contents were 1.9- (*p* = 0.002, Figure 3C) and 34-fold (*p* < 0.001, Figure 3F), respectively, lower in preterm infants than in term infants. Peptide counts and abundance of Ig J-chain, lambda-chain and Ig kappa-chain did not differ between preterm and term infant gastric samples (*p* > 0.05, Table S2). Peptide counts of SC (f19–603 of PIgR) decreased 37% and 56% from human milk to the stomach for preterm and term infants, respectively (*p* < 0.05, Figure 4A,B). Peptide abundance of SC decreased 89% and 68% from human milk to the stomach for preterm and term infants, respectively (*p* < 0.05, Figure 4C,D).

### 3.4. pH

For preterm and term mother-infant samples, pH values in gastric samples were lower than those in human milk (*p* < 0.001, Figure S5). Neither milk nor gastric sample pH differed between preterm and term mother-infant pairs (*p* > 0.05, Table S2).

## 4. Discussion

The Ig concentrations in milk from preterm- and term-delivering mothers have been previously studied [34,35,36,37]. No study has compared the survival of Igs in the stomach or intestine between preterm and term infants. Our recent studies demonstrated that preterm infants partially degrade IgA but not IgG and IgM in the stomach [21,22]. A few studies have measured the survival of milk Igs to infant stool [17,38]. However, measuring Igs in infant stool samples does not accurately represent the biologically relevant survival of Igs within the upper GI tract, as Igs are exposed to proteases from the infant gastrointestinal tract as well as protein-fermenting colonic bacteria, which can degrade them before they are identified in stool [39]. To begin to address this lack of knowledge, the present study examined the stability of human milk Igs—total IgA (SIgA/IgA), total IgM (SIgM/IgM), IgG, and total SC (SC/SIgA/SIgM)—during gastric digestion in preterm and term infants.

Concentrations of Igs in human milk from mothers delivering prematurely and at term were congruent with those found in previous studies [36,37,40,41]. Concentrations of total IgA or total SC did not differ between preterm and term milk, which matches with the observation in a previous study [34] that milk total IgA did not differ between preterm and term milk (3.2 mg/mL) from 6 to 28 days postnatal age. Ballabio et al. [34] observed a higher concentration of total IgA than measured in this present study and did not detect IgM or IgG in preterm and term milk (we detected IgM and IgG). This difference could be due to their use of an immunoelectrophoretic technique (SDS-PAGE and immunoblotting) to determine the concentration of Igs in their milk samples. ELISA is more quantitative (lower detection limit) than SDS-PAGE and immunoblotting. We found that total IgA concentration decreased with increasing postnatal age, PMA, and BW_s_ in preterm milk, which matches with this same previous study [34] (total IgA concentration decreased from colostrum to mature milk from preterm-delivering mothers). A few studies found higher amounts of total IgA in colostrum from preterm-delivering mothers compared with colostrum from term-delivering mothers [3,34,35,42]; however, as no colostrum was collected in the present study, we cannot compare our results to that data. Another study found that preterm milk IgA concentration was 1.1- to 1.4-fold higher than in term milk from 3 to 15 days but found no differences in concentration from 28 to 56 days of postnatal age [3]. Thus, the observed lack of difference for total IgA concentration between preterm and term milk in the present study is likely due to the older postnatal age (average: 39 days for preterm and 23 days for term infants) for milk collection.

The concentration of total SC (mostly SIgA but can include SC and SIgM) represented 73.5% of total IgA in preterm milk and 63.6% of total IgA in term milk. These percentages are somewhat lower than the observations reported by Goldman et al. [4] that total SC (called “SIgA” by the authors but actually representing total SC, as an anti-SC primary antibody was used) concentration represented 90% of the total IgA in term milk.

The concentration of IgG and total IgM were lower in human milk from preterm-delivering mothers than term-delivering mothers. Previous studies observed IgG and IgM concentrations in human milk that were similar between mothers delivering prematurely and at term [36,41]. On the other hand, Chandra et al. [3] found that IgM and IgG concentrations were 1.5-fold higher in preterm milk than in term milk from 14 to 28 days of postnatal age.

The total IgA concentration decreased from human milk to the preterm and term infant stomach. The stability of SC/SIgA/SIgM during gastric digestion in preterm suggests that SC remains intact and detectable by the anti-SC even if it is released from the SIgA complex. Unlike in preterm infant, total SC concentration decreased in the term infant stomach. The reduction of total IgA (SIgA/IgA) was likely due to degradation by proteases (pepsin and/or milk proteases [21]) and not acid-induced structural deterioration in the stomach, as incubation of standard IgA in acid conditions did not decrease its concentration (Figure S6). If we consider that 74% of total IgA in preterm milk is SIgA—we could not distinguish between SC/SIgA/SIgM, the decrease of total IgA likely derived from preterm gastric digestion of partly IgA and partly SIgA. We found higher peptide abundance of Ig alpha-chain (which could derive from either IgA or SIgA) in term infant gastric contents than in preterm infants, which suggests that IgA/SIgA is more digested by term infants than preterm infants.

We also observed that human milk total IgM decreased and IgG tended to decrease in the term stomach (48% and 49% reduction, respectively) but were stable in the preterm stomach. We found higher peptide counts and abundance of Ig mu (which could derive from either IgM or SIgM) and Ig gamma (which derive from IgG) in term infant gastric infants than preterm infants, which suggests that SIgM/IgM and IgG is more digested by term infants than preterm infants. This decrease of Igs is unlikely to relate to gastric pH as preterm and term infants’ gastric pH did not differ. We also observed that pH 4.5 had no effect on the stability of Igs (Figure S2). Gastric proteases are likely responsible for observed differences, as we previously demonstrated that gastric pepsin activity and proteolysis were higher in term infants than in preterm infants [20].

We observed that the stability of IgG and total IgM during gastric preterm digestion was higher than total IgA. No previous study evaluated the digestibility of Igs in the preterm or term infant stomach or small intestine, but a previous study [17] found a greater reduction of IgA compared with IgG or IgM in preterm infant stools. When preterm infants (1–28 days of postnatal age (GA unknown) 0.8–2 kg BW) were fed only infant formula or infant formula plus pasteurized pooled human milk supplemented with 600 mg daily of serum-derived human IgA (73%) and IgG (26%), the stool samples collected contained 1–10 mg IgG per g of dried feces (percentage reduction not calculated) and no IgA [17]. We showed that IgG was decreased in the term infant stomach but stable in the preterm infant stomach.

Using peptidomics, we demonstrated partial digestion (demonstrated by an increase in peptide counts and abundances from milk to the stomach) of Ig alpha-chain (from IgA and SIgA), Ig gamma-chain (from IgG), Ig mu-chain (from IgM or SIgM), and Ig J-chain (from IgA, SIgA, IgM or SIgM), Ig kappa-chain and Ig lambda-chain (from all Igs) in both preterm and term infants. Dallas et al. [43] also observed that alpha-1-chain (named IGHA1) was not detected in term milk but was detected in the term stomach. SC peptide counts decreased in both preterm and term gastric samples, suggesting that SIgA or SIgM were partially digested in the stomach from both infants. We found a decrease in peptide counts and abundance of SC (which could derive from either SIgA or SIgM) in both preterm and term infant gastric samples, suggesting a digestion of these Igs in the infant stomach.

A limitation of this study is the small number of samples for preterm and term infants. Another limitation is that we did not measure Ig concentrations in the infant intestine contents. Therefore, we plan to examine how human milk Ig concentrations change in the stomach and intestine in a future study with a larger number of preterm and term infants. The infants in our study had conditions that required a naso-gastric tube, which could affect Ig digestion. However, we included only infants with conditions that are not expected to interfere with protein digestion. Ideally, the study would include healthier infants; however, placing a feeding tube in infants that do not require them is very challenging from an ethical and patient enrollment perspective. Development of non-invasive techniques to collect digestive samples from fragile infants would be highly beneficial to this field. The placement of the nasogastric tube in these infants could also modify the gastric microbiome, which could influence Ig digestion. However, as we currently have no technique to assess gastric digestion in infants without a feeding tube, we cannot assess this potential impact.

## 5. Conclusions

The present study revealed that total IgA (SIgA/IgA) was digested in the preterm and term infant stomach. Human milk total SC (SC/SIgA/SIgM), IgM, and IgG were stable in the preterm stomach but were digested in the term stomach. Ig-derived peptides from all different Ig isotypes were in higher amounts in the gastric contents of term infants than in preterm infants, demonstrating a higher overall gastric Ig digestion in term infants. Therefore, human milk Igs are less digested in the preterm infant stomach than the term infant stomach. As the stomach represents only the beginning of the digestive system, the concentrations of human milk Igs in intestinal samples from preterm and term infants need to be determined to clarify their potential survival during infant digestion. The longer that milk Igs survive through the digestive system, the longer they can act as passive immune system components, which is particularly important in the context of the immune system immaturity of the early postnatal period in preterm and term infants.

## Figures and Tables

**Table 1:** Demographics of preterm- and term-delivering mother-infant pairs sampled for human milk and gastric contents at 2 h postprandial.

Demographics	Preterm-Delivering Mother Infant Pairs ^1–2^	Term-Delivering Mother-Infant Pairs ^1–2^
GA, weeks	27 ± 3 (23−32)	38.9 ± 0.5 (38−40)
Postnatal age, day	39 ± 28 (7−98)	23 ± 11 (16−42)
Postmenstrual age, day	32 ± 2 (30−37)	42 ± 2 (41−45)
Birth weight, kg	1.0 ± 0.4 (0.5−1.6)	3.5 ± 0.3 (3.0−3.8)
Infant sex	13 females; 2 males	6 females; 2 males
Mother’s age, year	35 ± 3 (32−39)	24 ± 10 (17−42)

^1^ Values are mean ± SD (range); ^2^ Number of paired milk and gastric samples from preterm and term infants is *n* = 15 and *n* = 8, respectively.

## Supplementary Materials

The following are available online at http://www.mdpi.com/2072-6643/10/5/631/s1, Table S1: Details for the assays performed in human milk and gastric samples; Table S2: Statistical results (*p*-values) for Wilcoxon matched-pairs signed rank test to compare immunoglobulin concentrations (ELISA) and peptides (peptidomics) in human milk and gastric samples in preterm and term infants (PM vs. PG or TM vs. TG); Table S3: Statistical results (*p*-value) from the linear regression of the pH and the concentration of total IgA (SIgA/IgA), total SC (SC/SIgA/SIgM), IgG and total IgM (SIgM/IgM) across postnatal age (P), gestational age at birth (GA), postmenstrual age (PMA), BW_b_, BW_s_ and feed volume (FV) in human milk and in gastric contents at 1, 2 and 3 h postprandial from preterm infants (23–32 week of gestational age, 7–98 days of postnatal age) and term infants (38–40 week of GA, 16–42 days of postnatal age); Figure S1: Immunoglobulin concentrations of (A) total IgM (SIgM/IgM) and (B) IgG in mother’s milks delivering prematurely (23–32 week of gestational age (GA), 7–98 days of postnatal age) and at term (38–40 week of GA, 16–42 days of postnatal age). Figures S2–S4: Peptide counts and abundance of human immunoglobulin fragments in human milk and gastric samples at 2 h postprandial time from paired mother-infant delivered prematurely (23–32 week of gestational age (GA), 7–98 days of postnatal age) and at term (38–40 week of GA, 16–42 days of postnatal age). Figure S5: The pH in human milk and gastric samples in human milk and gastric samples at 2 h postprandial time from paired (A) mother-infant delivered prematurely (23–32 week of gestational age (GA), 7–98 days of postnatal age) and (B) at term (38–40 week of GA, 16–42 days of postnatal age). Figure S6: Stability of standard immunoglobulins (Igs) before and after the incubation at pH 4.5.
