# Supplementary material for: Comparison of Human Milk Immunoglobulin Survival during Gastric Digestion between Preterm and Term Infants
Source: Nutrients. 2018 May 17;10(5):631. doi: 10.3390/nu10050631 (PMC5986510; doi:10.3390/nu10050631)
Supplement: Supplementary file 1 [file nutrients-10-00631-s001.pdf]

nutrients

MDPI

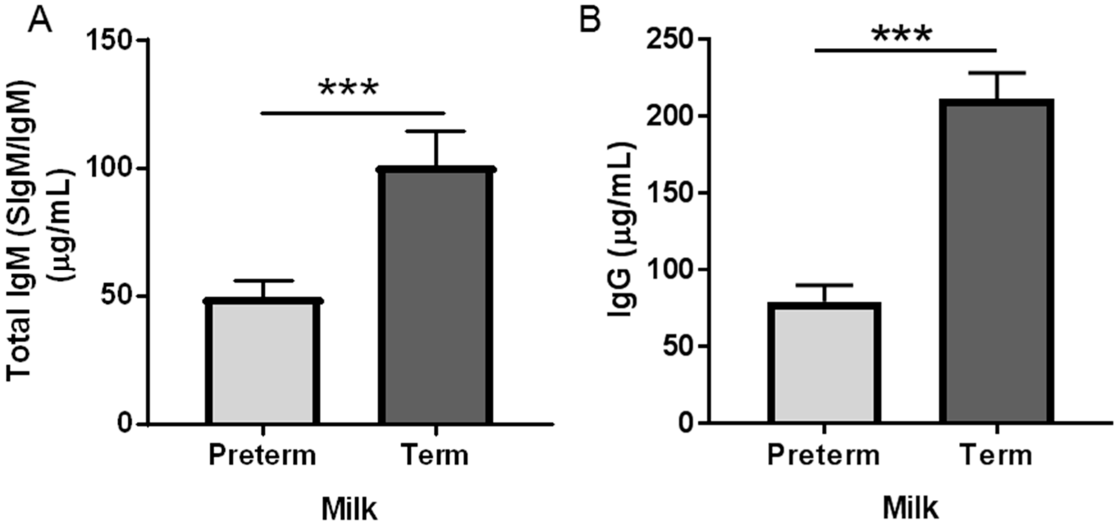

Figure S1. Immunoglobulin concentrations of (A) total IgM (SIgM/IgM) and (B) IgG in mother's milks delivering prematurely (23–32 week of gestational age (GA), 7–98 days of postnatal age) and at term (38–40 week of GA, 16–42 days of postnatal age). Values are mean ± SEM, $n = 15$ for preterm infants and $n = 8$ for term infants. Asterisks show statistical significant differences between variables (*** $p < 0.001$; * $p < 0.05$) using Mann Whitney test (unpaired samples for preterm vs. term).

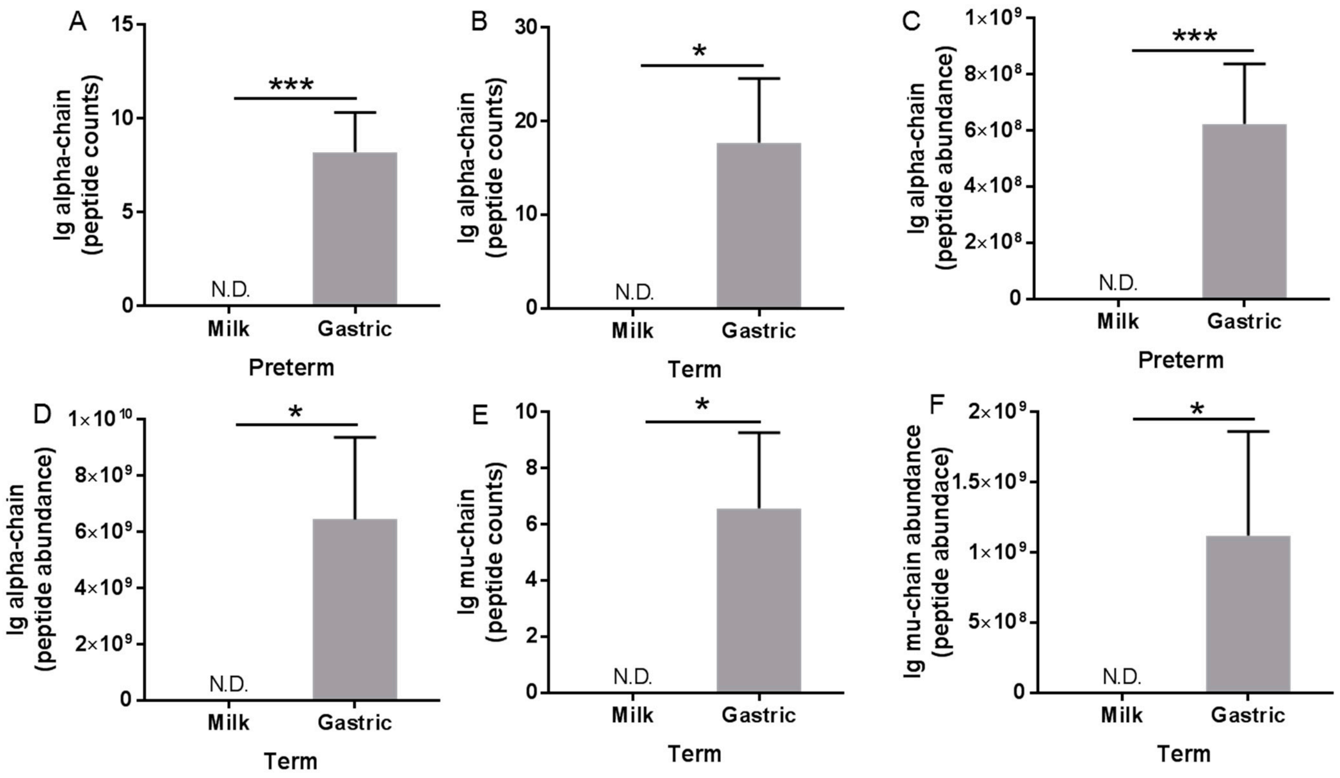

Figure S2. Peptide counts and abundance of human immunoglobulin fragments in human milk and gastric samples at 2 h postprandial time from paired mother-infant delivered prematurely (23–32 week of gestational age (GA), 7–98 days of postnatal age) and at term (38–40 week of GA, 16–42 days of postnatal age). Values are mean ± SEM, $n = 15$ for preterm infants and $n = 8$ for term infants. (A, B, C and D) Ig alpha-chain (from SIgA/IgA), (E and F) Ig mu-chain (from SIgM/IgM). Asterisks show statistical significant differences between variables (*** $p < 0.001$; * $p < 0.05$) using Wilcoxon matched-pairs signed rank test (paired samples for comparison in same infant).

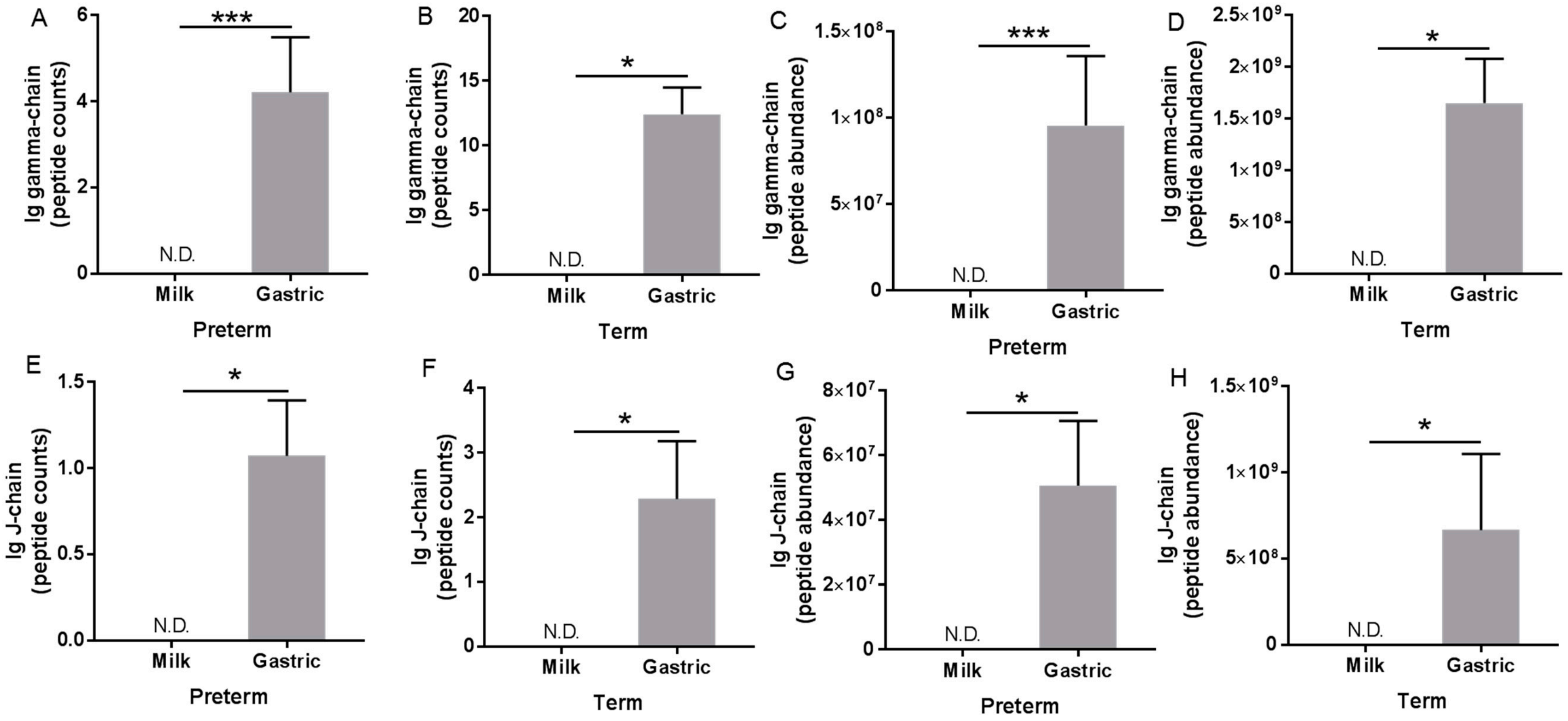

Figure S3. Peptide counts and abundance of human immunoglobulin fragments in human milk and gastric samples at 2 h postprandial time from paired mother-infant delivered prematurely (23–32 week of gestational age (GA), 7–98 days of postnatal age) and at term (38–40 week of GA, 16–42 days of postnatal age). (A, B, C and D) Ig gamma-chain (from SIgA/IgA), (E, F, G and H) Ig J-chain (from SIgA/IgA/SIgM/IgM). Values are mean ± SEM, n = 15 for preterm infants and n = 8 for term infants. Asterisks show statistical significant differences between variables (*** $p < 0.001$; * $p < 0.05$) using Wilcoxon matched-pairs signed rank test (paired samples for comparison in same infant).

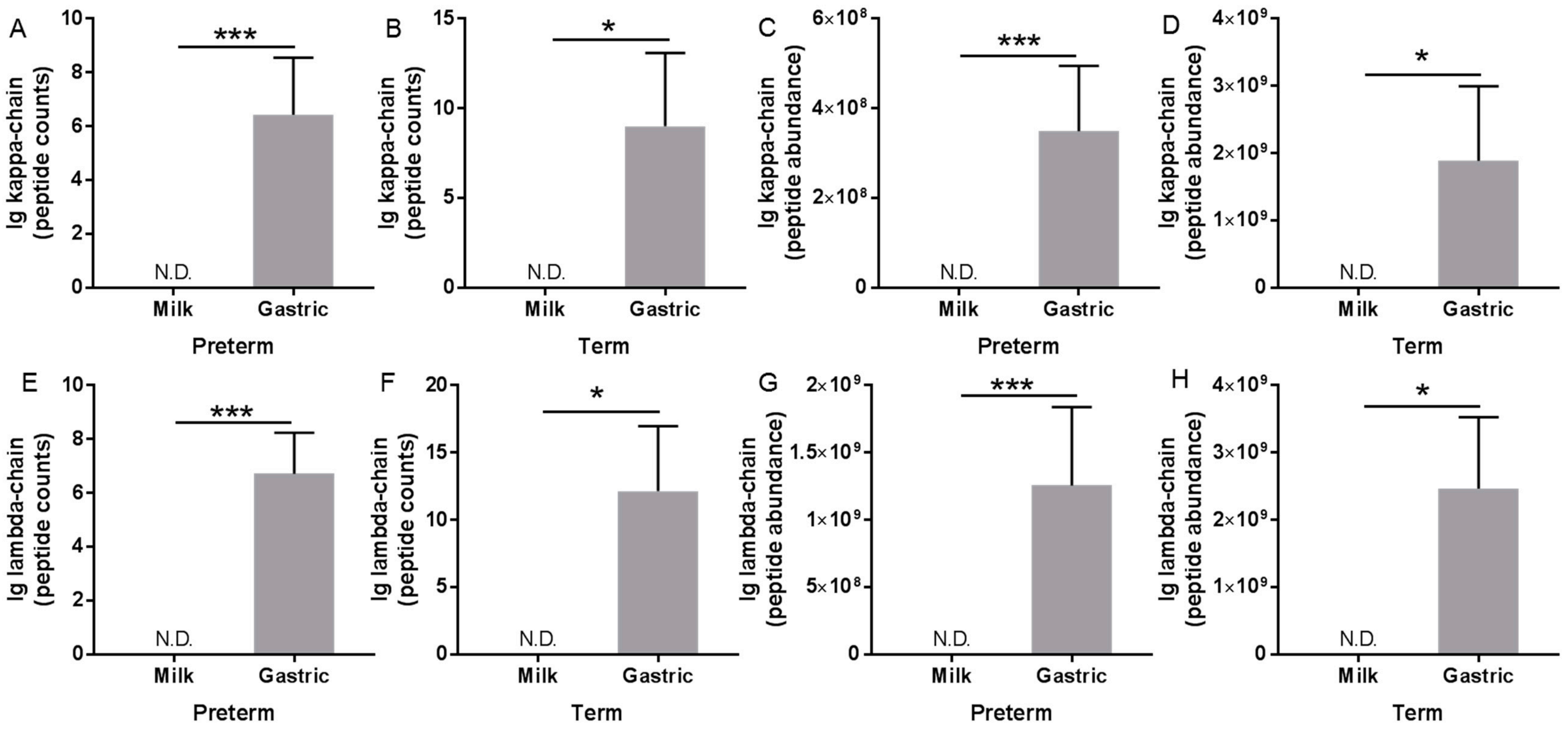

Figure S4. Peptide counts and abundance of human immunoglobulin fragments in human milk and gastric samples at 2 h postprandial time from paired mother-infant delivered prematurely (23–32 week of gestational age (GA), 7–98 days of postnatal age) and at term (38–40 week of GA, 16–42 days of postnatal age). Values are mean ± SEM, $n = 15$ for preterm infants and n = 8 for term infants. (A, B, C and D) Ig kappa-chain (from SIgA/IgA/SIgM/IgM/IgG), (E, F, G and H) Ig lambda-chain (from SIgA/IgA/SIgM/IgM/IgG). Values are mean ± SEM, $n = 15$ for preterm infants and $n = 8$ for term infants. Asterisks show statistical significant differences between variables (*** $p < 0.001$; * $p < 0.05$) using Wilcoxon matched-pairs signed rank test (paired samples for comparison in same infant).

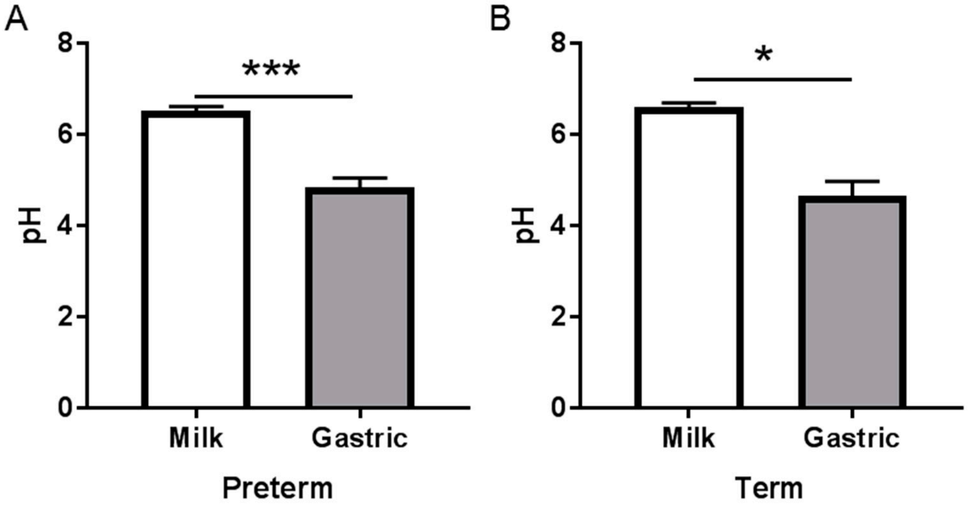

Figure S5. The pH in human milk and gastric samples in human milk and gastric samples at 2 h postprandial time from paired (A) mother-infant delivered prematurely (23–32 week of gestational age (GA), 7–98 days of postnatal age) and (B) at term (38–40 week of GA, 16–42 days of postnatal age). Values are mean ± SEM, $n = 15$ for preterm infants and $n = 8$ for term infants. Asterisks show statistical significant differences between variables (*** $p < 0.001$; * $p < 0.05$) using Wilcoxon matched-pairs signed rank test (paired samples for comparison in same infant).

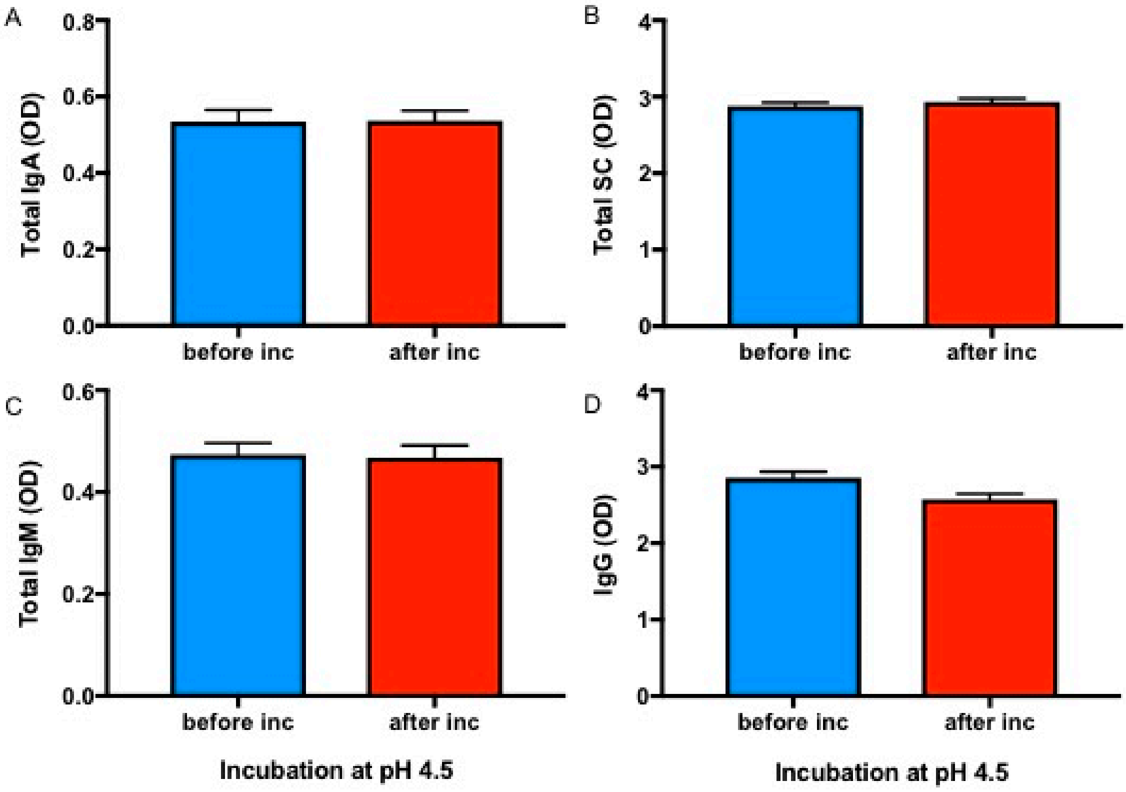

Figure S6. Stability of standard immunoglobulins (Igs) before and after the incubation at pH 4.5. (A) Concentrations of total IgA (SIgA/IgA), (B) total secretory component (SC/SIgA/SIgM), (C) total IgM and (SIgM/IgM) (D) IgG were determined by ELISA. No significant difference between groups ($p > 0.05$) was observed using paired Student t-tests. Values are mean ± SEM, $n = 3$.

Table S1. Details for the assays performed in human milk and gastric samples.

Type of assay	ELISA kit	Sample amount (µL)	Sample in dilution buffer	Standard curve
Human total IgA	IHUIGAKT, I.R.I [1]	4	1:4,000	0.1–100 ng/mL
Human secretory component (SC)	EKC35479, Biomatik	4	100	20–0.078 µg/mL
Human IgG	IHUIGGKT, I.R.I	4	1:100,000	0.2–200 ng/mL
Human IgM	IHUIGMKT, I.R.I	4	1:10,000	0.5–200 ng/mL

[1] Innovative Research, Inc.

Table S2. Statistical results (*p*-values) for Wilcoxon matched-pairs signed rank test to compare immunoglobulin concentrations (ELISA) and peptides (peptidomics) in human milk and gastric samples in preterm and term infants (PM vs PG or TM vs TG). Mann Whitney test to compare measurements of preterm and term infant samples in human milk or gastric samples (PM vs. TM and PG vs. TG). All tests were using nonparametric tests as the D 'Agostino & Pearson normality test did not pass for some groups. Paired milk and gastric samples were collected from preterm infants (23–32 wk of gestational age, 7–98 days of postnatal age) and term infants (38–40 wk of GA, 16–42 days of postnatal age). Values are *p*-value for preterm (n = 15) and/or term (n = 7) infants. PM; preterm milk; PG; preterm gastric; TM, term milk; TG, term gastric. C, concentration; PC, peptide counts; PA, peptide abundance.

Immunoglobulin	PM vs. PG	TM vs. TG	PM vs. TM	PG vs. TG
C total IgA	0.001	0.016	0.62	0.73
C total SC	0.33	0.031	0.18	0.17
C total IgM	0.54	0.016	<0.001	0.56
C IgG	0.25	0.026	<0.001	0.58
PC Ig alpha	<0.001	0.016	>0.99	0.081
PA Ig alpha	<0.001	0.016	>0.99	0.038
PC Ig gamma	<0.001	0.016	>0.99	0.002
PA Ig gamma	<0.001	0.016	>0.99	<0.001
PC Ig mu	0.12	0.031	>0.99	0.032
PA Ig mu	0.12	0.031	>0.99	0.010
PC Ig J	0.016	0.031	>0.99	0.20
PA Ig J	0.016	0.031	>0.99	0.052
PC Ig lambda	<0.001	0.016	>0.99	0.39
PA Ig lambda	<0.001	0.016	>0.99	0.12
PC Ig kappa	0.001	0.031	>0.99	0.45

PA Ig kappa	0.001	0.031	>0.99	0.29
PC SC	0.001	0.016	0.64	0.52
PA SC	0.006	0.031	0.009	0.25
pH	< 0.001	< 0.001	< 0.001	< 0.001

Table S3. Statistical results (*p*-value) from the linear regression of the pH and the concentration of total IgA (SIgA/IgA), total SC (SC/SIgA/SIgM), IgG and total IgM (SIgM/IgM) across postnatal age (P), gestational age at birth (GA), postmenstrual age (PMA), BW_b, BW_s and feed volume (FV) in human milk and in gastric contents at 1, 2 and 3 h postprandial from preterm infants (23–32 wk of gestational age, 7–98 days of postnatal age) and term infants (38–40 wk of GA, 16–42 days of postnatal age). Values are *P*-value for preterm (*n* = 15) and/or term (*n* = 7) infants. Pearson correlation coefficient (*r*) and slope (*s*) are included when the *p*-value is < 0.1. PM, preterm milk; PG, preterm gastric; TM, term milk; TG, term gastric; C, concentration; PC, peptide counts; PA, peptide abundance.

**Immunoglobulin**	**Factor**	**PM**	**PG**	**TM**	**TG**
C Total IgA	P	0.002 (*r* = –0.74, *s* = –13)	0.021 (*r* = –0.59, *s* = –3.4)	0.77	0.20
	GA	0.051 (*r* = 0.51, *s* = 0.15)	0.17	0.28	0.83
	PMA	0.032 (*r* = -0.55, *s* = –115)	0.059 (*r* = –0.50, *s* = –35)	0.50	0.35
	BW_b	0.097 (r = 0.44, s = 559)	0.23	0.075 (*r* = -0.71, *s* = –480)	0.43
	BW_s	0.007 (*r* = -0.70, *s* = –843)	0.043 (*r* = -0.57, *s* = –223)	NA	NA
	FV	0.059 (*r* = 0.52, *s* = 20)	0.17	0.92	0.92
C total SC	P	0.28	0.51	0.50	0.073 (*r* = –0.77, *s* = –5.3)
	GA	0.25	0.42	0.58	0.76
	PMA	0.80	0.92	0.66	0.18
	BW_b	0.20	0.46	0.28	0.17
	BW_s	0.89	0.57	NA	NA
	FV	0.62	0.31	0.90	0.90
C total IgM	P	0.075 (*r* = –0.49, *s* = –0.4)	0.33	0.30	0.52
	GA	0.20	0.14	0.18	0.83

	PMA	0.18	0.89	0.92	0.11
	BW_b	0.46	0.19	0.26	0.50
	BW_s	0.032 (*r* = –0.60, *s* = –31)	0.51	NA	NA
	FV	0.012 (*r* = 0.65, *s* = 1.4)	0.021 (*r* = 0.61, *s* = 1.6)	0.25	0.66
C IgG	P	0.23	0.22	0.61	0.84
	GA	0.090 (*r* = -0.45, *s* = –5.7)	0.91	0.65	0.88
	PMA	0.84	0.014 (*r* = –0.62, *s* = –16)	0.16	0.36
	BW_b	0.065 (*r* = –0.49, *s* = –51)	0.95	0.68	0.016 (*r* = 0.80, *s* = 278)
	BW_s	0.88	0.026 (r = -0.61, s = -85)	NA	NA
	FV	0.23	0.72	0.53	0.53
